# Optimization of fecal near infrared spectroscopy for predicting organic matter digestibility in cows using local algorithms

**DOI:** 10.1093/jas/skag154

**Published:** 2026-05-15

**Authors:** Donato Andueza, Cécile Martin, Marion Brandolini-Bunlon, Nadège Edouard, Peter Lund, Christopher K Reynolds, Les A Crompton, Eric Froidmont, Isabelle Morel, Pierre Nozière, Gonzalo Cantalapiedra-Hijar

**Affiliations:** Université Clermont Auvergne, INRAE, VetAgro Sup, UMR Herbivores, Saint-Genès-Champanelle F-63122, France; Université Clermont Auvergne, INRAE, VetAgro Sup, UMR Herbivores, Saint-Genès-Champanelle F-63122, France; Université Clermont Auvergne, INRAE, UNH, Plateforme d’Exploration du Métabolisme, MetaboHUB Clermont, Clermont-Ferrand F-63000, France; PEGASE, INRAE, Institut Agro, Saint-Gilles 35590, France; Department of Animal and Veterinary Sciences, Aarhus University, PO Box 50, Tjele DK-8830, Denmark; Centre for Dairy Research, Department of Animal Sciences, School of Agriculture, Policy, and Development, University of Reading, Reading RG6 6EU, United Kingdom; Centre for Dairy Research, Department of Animal Sciences, School of Agriculture, Policy, and Development, University of Reading, Reading RG6 6EU, United Kingdom; Walloon Agricultural Research Center (CRA-W), Gembloux B-5030, Belgium; Agroscope, Ruminant Nutrition and Emissions, Posieux CH-1725, Switzerland; Université Clermont Auvergne, INRAE, VetAgro Sup, UMR Herbivores, Saint-Genès-Champanelle F-63122, France; Université Clermont Auvergne, INRAE, VetAgro Sup, UMR Herbivores, Saint-Genès-Champanelle F-63122, France

**Keywords:** alternative, animal welfare, cattle, nutritive value, proxy

## Abstract

Accurate estimation of diet digestibility is essential for the efficient and sustainable production of milk and meat by ruminants. However, standard in vivo methods for assessing the nutritive value of ruminant feed are both time-consuming and costly. As a result, indirect methods based on the infrared absorbance of animal feces have been developed as practical alternatives. Most existing prediction models rely on global calibrations, which establish relationships between organic matter digestibility (OMD) and near-infrared (NIR) spectra across an entire sample population. However, using only a subset of samples, those most similar to the target sample, may improve prediction accuracy. This study compares a global prediction approach, partial least squares regression (PLSR), with several Local modeling techniques: Locally weighted PLS regression (LWPLSR), k-nearest neighbors Locally weighted PLS regression (KNN-LWPLSR), and an aggregated version of the latter (KNN-LWPLSR-AGG), for predicting OMD in cattle. A dataset of 466 fecal samples with corresponding in vivo OMD measurements was used. Of these, 299 samples were used for model calibration, while the remaining 167 were split into two groups: 76 for external validation and 91 for testing under routine conditions. Results showed no significant difference in prediction accuracy among the Local methods (*P* > 0.05). However, LWPLSR outperformed the global PLSR model (*P* < 0.05). The standard error of the in vivo standard reference method was estimated at 0.013 g/g, while the best NIR-based prediction error was 0.016 g/g. Given its balance between predictive accuracy and computational efficiency, LWPLSR is recommended for practical applications.

## Graphical abstract

**Graphical Abstract:**
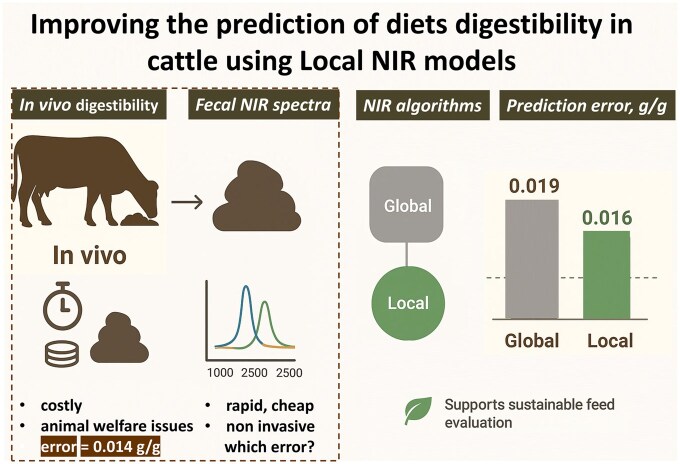

## Teaser

The utilization of some Local models to predict the organic matter digestibility in cattle, based on fecal near-infrared spectra, yielded superior results in comparison to those attained through the application of a global prediction model.

## Introduction

Current livestock production systems must balance economic efficiency with the nutritional adequacy of animals to ensure long-term sustainability. Achieving this requires minimizing feed costs while ensuring that animals receive sufficient nutrients for growth, reproduction, and maintenance. Accurate assessment of organic matter digestibility (OMD) is essential for optimizing animal performance, as diet digestibility determines the proportion of nutrients that animals can absorb and utilize for physiological functions. OMD, a key determinant of energy availability for maintenance and animal production (INRA 2018), is typically determined through in vivo measurements conducted under standardized conditions (Mesgaran et al. 2020). However, such trials are becoming increasingly difficult to conduct due to animal welfare issues (Veissier et al. 2025) and are often impractical due to their high demands in terms of time, labor, and cost (Bellagi et al. 2025). To address these limitations, various laboratory-based methods have been developed to estimate OMD. These include in vitro techniques that simulate digestive processes using rumen fluid inoculum (Tilley and Terry 1963; Menke et al. 1979) or cellulolytic enzymes (Aufrère and Michalet-Doreau 1988). In recent decades, near-infrared reflectance spectroscopy (NIRS) has emerged as a powerful tool for predicting the nutritive value of diets using dried forage samples (Andueza et al. 2011) and feces (Boval et al. 2004; Decruyenaere et al. 2009). Unlike feed, fecal samples provide a non-invasive means of estimating OMD at the individual animal level, which might be particularly useful for precision feeding strategies aimed at tailoring diet composition to the specific nutritional requirements of each animal.

The conventional approach for developing NIRS calibration models is partial least squares (PLS) regression, which performs well under the assumption of a linear relationship between spectral data and reference values (Höskuldsson 1988; Wold et al. 2001). However, in the case of heterogeneous samples, non-linear relationships often arise between spectral absorbance and chemical composition, reducing the accuracy of global linear models (Shenk et al. 1997). To improve model robustness, it is recommended that the calibration dataset capture all sources of variability expected during prediction (Shenk et al. 1997). While this can increase model generalizability, it may also reduce predictive accuracy when the dataset is large and/or includes highly diverse sample types (Fernández-Cabanás et al. 2023). Fecal material, like many agricultural products, is inherently heterogeneous, which complicates the establishment of global linear predictive models (Lesnoff et al. 2020). In such contexts, Local regression approaches—such as Local PLS regression models—may offer improved accuracy. These methods generate a tailored model for each sample by selecting a subset of similar samples from a larger calibration database (Berzaghi et al. 2000). Lesnoff et al. (2020) emphasized that Local averaging methods could be particularly advantageous when working with heterogeneous biological materials common in agricultural settings.

The aim of this study was to evaluate whether Local regression methods (Locally weighted PLS regression [LWPLSR], k-nearest neighbors Locally weighted PLS regression [KNN-LWPLSR], and k-nearest neighbors Locally weighted PLS regression with aggregation [KNN-LWPLSR-AGG]) outperform conventional global PLS regression models in predicting dietary OMD from the NIR spectra of bovine feces. A secondary objective was to compare the performance of Local methods mentioned above and to estimate the prediction error associated with each individual OMD value predicted by the different methods and to conduct a statistical power analysis to illustrate the strengths and limitations of these prediction models.

We hypothesized that Local approaches would yield higher predictive accuracy than global PLS regression, and that among Local strategies, average PLS Local regression would be particularly well-suited compared to other methods for estimating OMD in cattle based on fecal NIR spectra.

## Material and methods

The experimental protocols of all trials included in our database were approved by national ethical committees as described in their respective scientific publications. For the unpublished trials used in this study, the experimental protocols were also approved by the national animal ethics committee (approval numbers: APAFIS #16174-2018071817132587-v2 and APAFiS #5686-201606132002401 v3 for trials 10 and 11 of the calibration subset, respectively [Table 1], and APAFIS#8518-2016120622288477 v3 for trial 1of the test subset [Table 1]). The trials number 4 of the calibration subset and the number 2 of the test subset (Table 1; Reynolds et al. 2016) were approved and monitored under the UK Home Office Animals (Scientific Procedures) Act 1986 (Project Licence PA75D3A9E) and by the University of Reading’s Home Office Animals Procedures Committee.

**Table 1:** Description of the experimental trials used in this study.

Trial	Type of animal (breed)	Type of diet	Number of animals	Number of fecal samples (Cal + Val KS)	Experimental design	Publication	Experimental length	Drying and grinding method
**Calibration and validation subsets**								
**1**	Beef cows (CH)	Hay or corn silage+ concentrates	16	33 + 31	2 successive periods of digestibility (1 period per aliment)	de la Torre et al. (2019)	6 d	60 °C 72 h 1 mm HM
**2**	Dairy cows (H)	Grass + corn silage + concentrates	6	6 + 6	Cross-over design	Herremans et al. (2020)	5 d	60 °C 72 h 1 mm CM
**3**	Dairy cows (H)	Corn silage + hay + dehydrated lucerne + concentrates	4	9 + 6	Latin square	Fanchone et al. (2013)	6 days	60 °C 48 h 1 mm HM
**4**	Dairy cows (H)	Corn silage + grass + concentrates	24	58 + 13	Continuous—weeks 6, 20 and 34 of lactation	Reynolds et al. (2016)	5 days	60 °C 72 h 1 mm CM
**5**	Dairy cows (H)	Corn silage+grass silage + hay + concentrates	8	28 + 3	2 Latin squares	Mendowski et al. (2019)	6 days	60 °C 72 h 1 mm HM
**6**	Dairy cows (H)	Corn silage+grass silage + hay + concentrates	16	28 + 4	2 Latin squares	Mendowski et al. (2020)	6 days	60 °C 72 h 1 mm HM
**7**	Dairy cows (H)	Hay + concentrates	4	13 + 3	Latin square	Martin et al. (2021) *	5 days	60 °C 48 h 1 mm HM
**8**	Dairy cows (H)	Grass silage + hay + concentrates	8	28 + 4	Latin square	Martin et al. (2023) *	5 days	60 °C 48 h 1 mm HM
**9**	Dairy cows (H)	Corn silage + hay + concentrates	16	30 + 1	2 groups of 8 animals	Guyader et al. (2016)	5 days	60 °C 48 h 1 mm HM
**10**	Dairy cows (H)	Corn silage + concentrates	8	27 + 4	2 Latin squares	Unpublished	4 days	60 °C 72 h 0.8 mm HM
**11**	Dairy cows (H)	Corn silage + dehydrated lucerne + concentrates	4	15 + 1	Latin square	Unpublished	4 days	60 °C 48 h 0.8 mm HM
**12**	Dairy cows (H)	Grass silage + hay + concentrates or corn silage + concentrates	8	24	2 Latin squares	Bougouin et al. (2018) *; Bougouin et al. (2019)*	5 days	60 °C 72 h 1 mm HM
**Test subset**								
**1**	Dairy cows (H)	Corn silage + concentrates	6	23	Cross-over design	Unpublished	4 days	60 °C 72 h 0.8 mm HM
**2**	Dairy cows (H)	Corn silage+ grass+concentrates	12	36	Continuous—weeks 6, 20 and 34 of lactation	Reynolds et al. (2016)	5 days	60 °C 72 h 1 mm CM
**3**	Dairy cows (H)	Corn silage + grass silage + hay + concentrates	16	32	2 Latins square	Manzocchi et al. (2023)	6 days	60 °C 72 h 1 mm CM

* In these trials, fecal samples contained urine. H = Holstein; CH = Charolais; HM = Hammer mill; CM = Cyclonic mill. Cal = calibration subset. Val KS = validation subset according Kennard Stone algorithm.

A total of 466 fecal samples, collected from 14 different *in vivo* digestibility trials conducted in cattle, were used in this study. A brief overview of each trial is provided in Table 1. The trials were conducted across four research centers located in three countries: France, Belgium, and the United Kingdom. The dataset includes both beef (*n* = 16) and dairy (*n* = 140) cows, representing a range of ages and physiological stages. Digestibility trials were performed in vivo following the methodology described by Mesgaran et al. (2020). The OMD was calculated using measurements of dry matter and ash content from the diet offered, the feed refusals, and the feces according to the following equation:


OMD (%)=(OM intake – OM in feces)×100/OM intake

Where OMD is the organic matter digestibility and OM is organic matter.

The mean, minimum and maximum values of OMD in our database were 0.709 g/g, 0.597 g/g, and 0.782 g/g, respectively (Table 2). For each digestibility measurement period, a representative composite sample of the total fecal output was collected from each animal and subsequently used for NIR spectral acquisition. In general, on a daily basis, the total feces output was homogenized and a sample was obtained and stored in a frozen state. After the digestibility trial, the samples were thawed, and a representative composite sample was prepared based on the daily fecal output.

**Table 2:** Descriptive statistics for organic matter digestibility (g/g) of the diet within calibration validation and test subsets used for fecal near infrared spectroscopy modelling.

	N	Mean	Median	Min	Max	SD	CV
**Calibration subset**	299	0.709	0.715	0.597	0.782	0.0350	4.94
**Validation subset**	76	0.699	0.708	0.631	0.770	0.0384	5.52
**Test subset**	91	0.716	0.717	0.659	0.760	0.0241	3.37

N: number of samples; Min: Minimum value; Max: Maximum value; SD: Standard deviation; CV: coefficient of variation;

### Acquisition of the near-infrared spectroscopy spectra

After homogenization, dried at 60 °C during 48–72 h and ground at 0.8–1 mm screen using a cyclonic or hammer mill (Table 1), representative composite fecal samples were placed in 50 mm diameter ring cups and scanned in reflectance mode using a Foss NIRSystems Model 6500 scanning Visible/NIR spectrometer (Foss NIRSystems, Silver Spring, MD, USA). Spectral data were collected at 2 nm intervals over the wavelength range of 400 to 2,500 nm using the ISIScan software. Each spectrum was obtained by averaging 32 consecutive scans to reduce signal noise. A reference scan, using the instrument’s internal ceramic reference tile, was performed immediately before and after each sample scan. Reflectance values were subsequently transformed into absorbance values using the equation:


absorbance=log(1/reflectance).

### Datasets

A principal component analysis was performed on the spectral data of the fecal samples to describe the datasets and analyze the data structure. In addition, several supplementary variables, both quantitative and qualitative, were reported. These variables included the type of forages and concentrates in each spectrum, as well as the forage/concentrate ratio, grind size, digestibility values, and the type of mill used for grinding (hammer mill or cyclonic mill).

**Figure 1:**
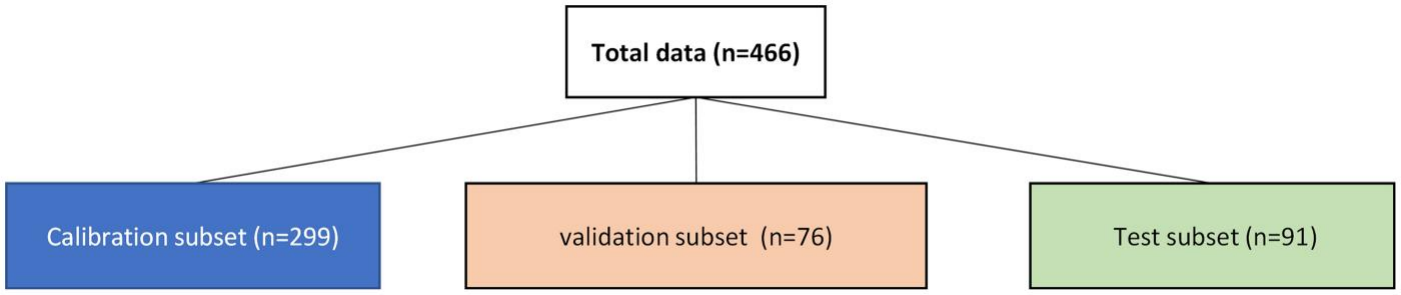
Schematic representation of data partitioning into calibration, external validation and test subsets.

Predicting models were developed using the R statistical software environment (R Core Team 2024). To evaluate model performance, the full dataset (*n* = 466) was divided into three subsets (#1 calibration, #2 validation, and #3 test; Table 1; Figure 1). The calibration subset was used to train models with 299 samples. The validation subset contained 76 samples and were selected using the Kennard–Stone algorithm (Kennard and Stone 1969). This algorithm maximizes sample diversity to ensure that both calibration and validation subsets are representative of the overall spectral variability while remaining distinct (ie validation subset contains samples obtained in the same conditions to those used in the calibration subset). The test subset was used for an external evaluation of the model, ensuring coverage of the spectral space and facilitating reproducibility. This subset, consisting of 91 samples (about 20% of the total available samples), was drawn from two independent Exp. and the third year of a third Exp. (representing field conditions different from those used in the calibration subset). This dual validation and test strategy allowed us to evaluate model performance both under conditions closely related to the calibration data (validation subset) and under more realistic, field-like conditions (test subset). During and after the calibration phase, and prior to applying models to the validation and test subsets, spectral similarity between each sample in both subsets and the centroid of the calibration subset was assessed using the standardized Mahalanobis distance (H). Following the criterion proposed by Shenk and Westerhaus (1994), samples with H-values greater than 3 were considered outliers and excluded from model evaluation. Four samples were thus removed from the calibration subset. No samples were removed from the validation and test subsets.

#### Calibration development

Prediction models were developed using four different regression techniques. All models were built using spectral data preprocessed with a first derivative transformation, according to the Savitzky–Golay method (Savitzky and Golay 1964), employing a third-order polynomial and a smoothing window of 11 spectral points. Additionally, standard normal variate transformation was applied as a scatter correction method (Barnes et al. 1989). Global PLS regression (Geladi and Kowalski 1986) was used as the reference method, as it is the most commonly applied technique when working with NIR spectral data due to the expected high collinearity among spectroscopic variables. Three Local modeling approaches described by Lesnoff et al. (2020, 2022) were evaluated: LWPLSR, KNN-LWPLSR, and KNN-LWPLSR-AGG.

**Figure 2:**
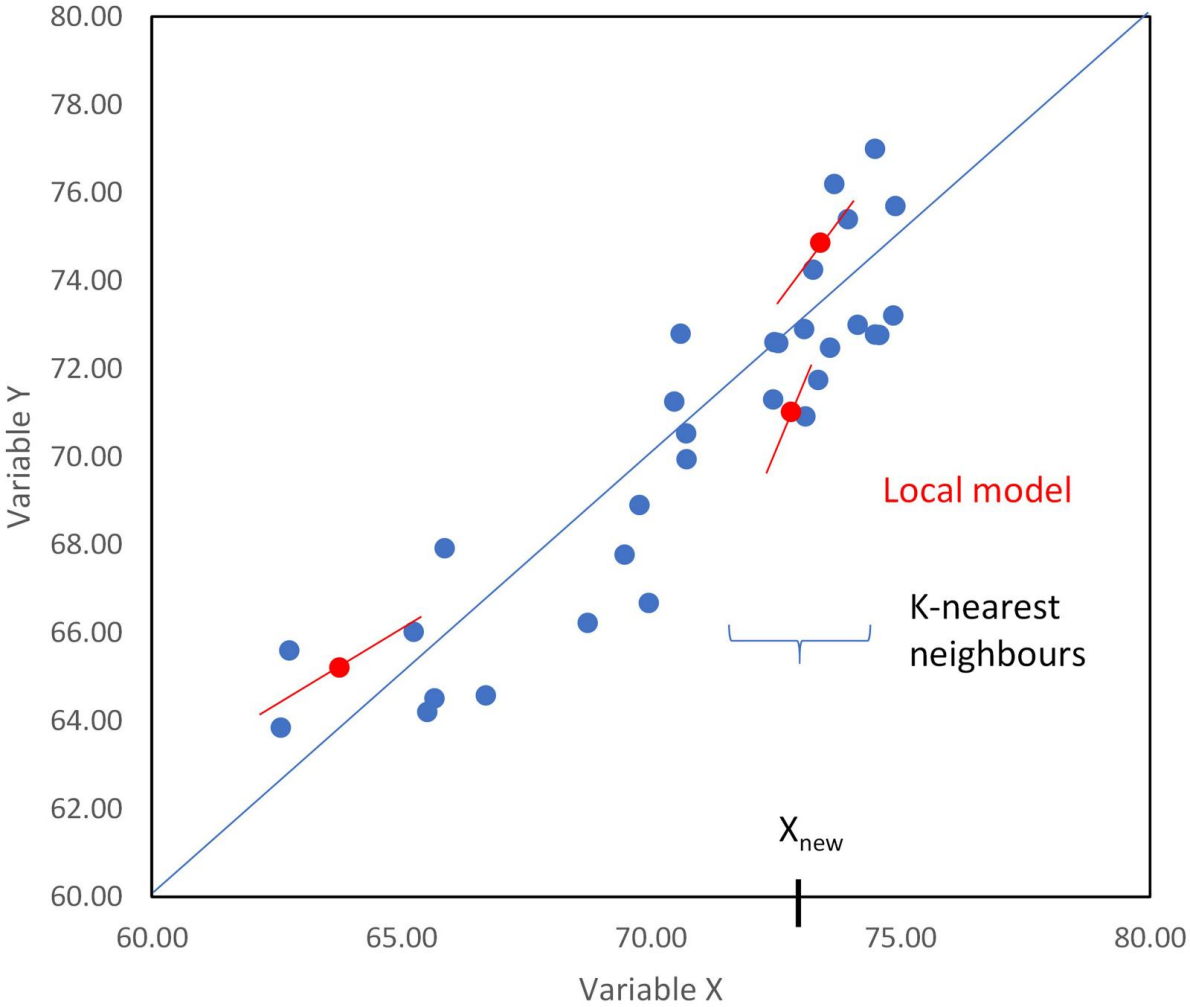
Illustration of the Local weighted regression principle. In order to predict the variable Y (OMD in this study) for each new sample (variable X_new_, red dots), a specific number of neighbors of the sample are selected from the database (K-nearest neighbors) in order to develop a sample-specific calibration model. Global model is represented as the blue regression line while Local models are depicted in red.

In Locally weight PLS regression approaches, a PLS regression model was developed individually for each sample to be predicted. Spectra similarities, based on the H-distance calculated in the latent variable space defined by a preliminary PLS regression, are computed between each sample of the data matrix and the target sample. Weights, derived from these similarities, are then applied in the PLS regression model (Figure 2). The closer a sample is to the prediction target in this space, the greater the weight it receives. In the LWPLSR approach, all samples of the data matrix are included in the final PLS regression model. In the KNN-LWPLSR variant, only a subset of the closest spectra is selected. The KNN-LWPLSR-AGG method extends this principle by performing multiple KNN-LWPLSR predictions using varying numbers of latent variables, then averaging the resulting predicted values to produce a final, aggregated prediction. The parameters optimized for the LWPLSR and KNN-LWPLSR models were the weight function parameter (h), which determines the influence of distance, and the number of nearest neighbors (k). For the KNN-LWPLSR-AGG model, additional parameters were optimized, including the minimum and maximum number of latent variables (nlv min and nlv max). The tested parameter values are provided in Table 3.

**Table 3:** Values tested for the parameters of the classic partial least square (PLS) regression, the Locally weighted PLS regression (LWPLSR), the k-nearest neighbors LWPLSR (KNN-LWPLSR) and the KNN-LWPLSR aggregated (KNN-LWPLSR-AGG).

Model	Nlv	Nlv min	Nlv max	h	k
**PLS**	1-30				
**LWPLSR**	1-30			0.5, 1, 2, 3, 4, 5, 6	
**KNN-LWPLSR**				0.5, 1, 2, 3, 4, 5, 6, inf	50, 100, 150, 200, 250
**KNN-LWPLSR-AGG**		1-5 each 1	15 to 30 each 5	0.5, 1, 2, 3, 4, 5, 6, inf	50, 100, 150, 200, 250

Nlv: number of latent variables; nlv min: minimal number of latent variables; Nlv max: minimal number of latent variables; h: defines the weight function form; k: number of neighbors; inf: infinity.

Model performance was initially evaluated using a five-fold interleaved cross-validation procedure. The primary metrics used for assessing cross-validation performance were the coefficient of determination in cross-validation (R^2^CV) and the root mean square error of cross-validation (RMSECV). Test performance was assessed using the coefficient of determination of prediction (R^2^P), root mean square error of prediction (RMSEP), bias, and RMSEP corrected for bias (RMSEP(C)). In addition, prediction errors were expressed relative to the mean observed values to facilitate comparison across models. Bias and RMSEP(C) values among the four models were compared following the method proposed by Fearn (1996): bias was evaluated using a paired-sample t-test with confidence intervals, and RMSEP(C) values were compared using confidence intervals for the ratio of standard deviations. All statistical analyses were performed using R software (version 2024.12.1).

### Reference method

The standard error of the reference method was estimated from the study of de la Torre et al. (2019), which involved four digestibility trials conducted on the same beef cows and was therefore suitable for inferring measurement error. Two consecutive trials were carried out under feeding a hay-based diet, and two under a corn silage and concentrate diet, using the same animals. In that trial, OMD was analyzed by a repeated measures mixed-effects model, specified as follows:


$$Y_{\text{ijkl}}=\mu +D_{i}+P_{j}+{\;A}_{k}+\varepsilon_{\text{ijkl}};$$

Where:

µ = overall mean;

D = diet effect (fixed)

P = period effect (fixed)

A = animal effect (random)

The uncertainty around individual predictions was estimated by calculating a 95% confidence interval for each OMD value using the formula:

±1.96 × RMSE, where RMSE represents the root mean square error of the model. This interval represents the minimum detectable difference of OMD, between two samples using this methodology.

### Power analysis

Finally, to assess the sensitivity of the developed models in detecting differences in OMD, we conducted a statistical power analysis. Specifically, we estimated the minimum detectable effect size given the error of the model (RMSEP), assuming a statistical power of 80% (1−β) and a Type I error rate (α) of 5%. The following formula, commonly used to calculate the minimum number of animals required to detect a statistically significant difference, was applied (Gupta et al. 2016):


$$\text{number}\;\text{of}\;\text{animals}=2\times {\left(Z_{\alpha }+{\;Z}_{\left[1-\beta \right]}\right)}^{2}\times \left({\text{SD}}^{2}/d^{2}\right)$$

where:

- Z_α_ + Z_[1-β]_ are the standard normal deviates corresponding to the Type I error and statistical power, respectively, where Z_α_ = 1.96 and Z_[1-β]_ = 1.28 when comparing two means,
- SD is the standard deviation and approached here by the RMSEP of the model,
- d is the effect size or the minimal difference in OMD intended to be detected.

## Results

The mean, median, standard deviation, minimum, and maximum values for the diet OMD for the calibration and validation subsets are presented in Table 2. Overall, the calibration and validation subsets covered similar ranges, and the mean, median, and standard deviation values were comparable (with coefficients of variation [CV] of 4.9% and 5.5%, respectively). However, the test subset was characterized by a narrower range and a lower standard deviation compared to the calibration and validation subsets (CV = 3.4%). Using data from de La Torre et al. (2019), one of the trials included in the present work, we calculated the standard error associated with the reference or gold standard method for digestibility, which was 0.0135 g/g. The descriptive statistics for the OMD were: number of OMD values = 64. Mean, median, standard deviation, minimum, and maximum values were 0.71 g/g, 0.71 g/g, 0.035 g/g, 0.60 g/g, and 0.78 g/g, respectively. The coefficient of variation was 4.94%.

**Figure 3:**
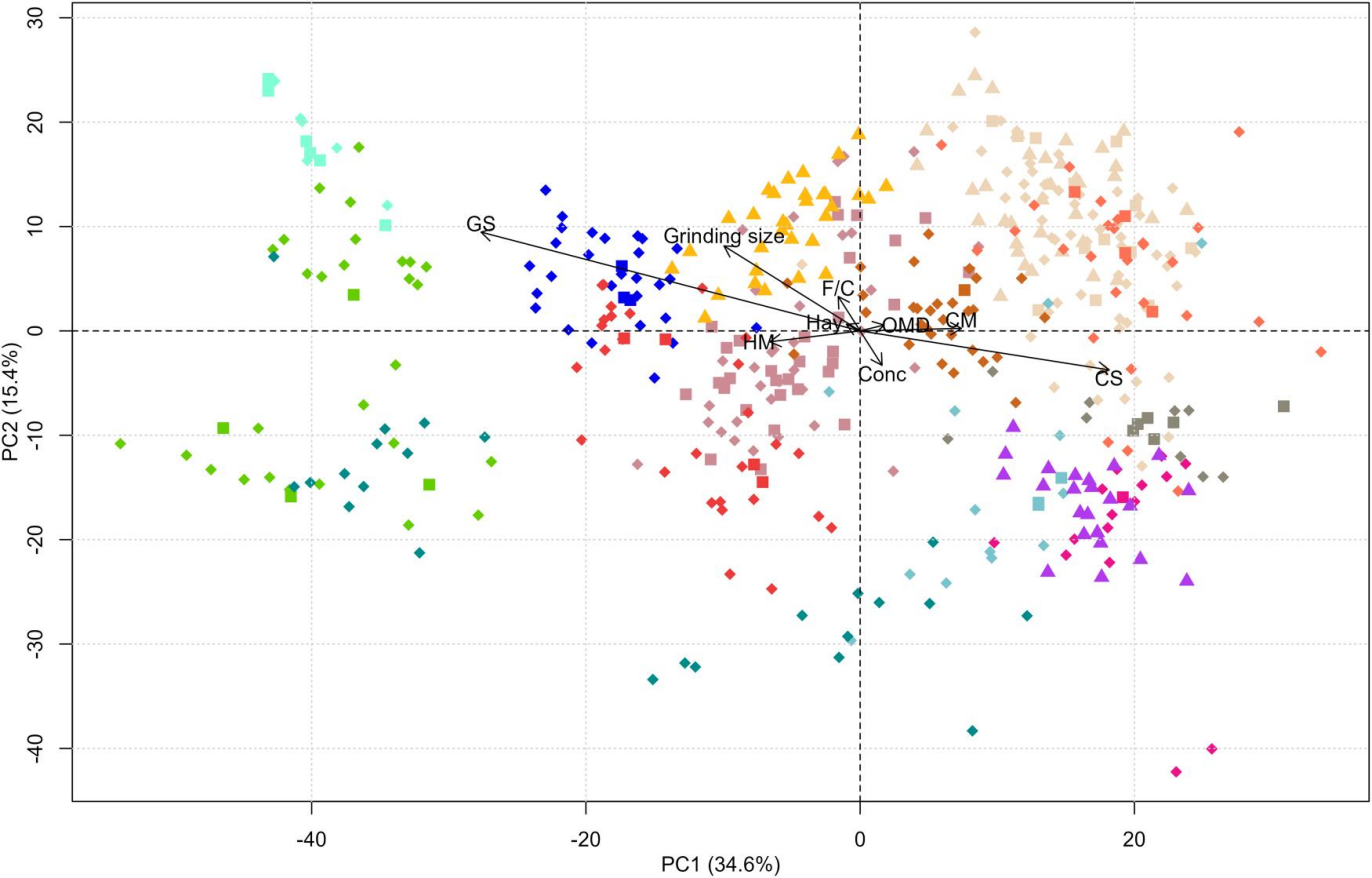
Representation of samples in the spectral database based on their scores on the first two principal components. Colors indicate the experiment from which the samples originated. Samples represented by ◇ belong to the calibration subset, whereas those represented by □ belong to the validation subset obtained using the Kennard–Stone method. Samples in the test subset are represented by △. The biplot also includes the following supplementary variables: grass silage (GS), hay, corn silage (CS), concentrate (Conc), forage-to-concentrate ratio (F/C), organic matter digestibility (OMD), grinding size, and milling type: hammer mill (HM) and cyclonic mill (CM). PC1 = Principal Component 1; PC2 = Principal Component 2. ◆■ Samples from the experiment by de la Torre et al. (2019). ◆■ Samples from the experiment by Herremans et al. (2020). ◆■Samples from the experiment by Fanchone et al. (2013). ◆■▲ Samples from the experiment by Reynolds et al. (2016). ◆■ Samples from the experiment by Mendowski et al. (2019). ◆■ Samples from the experiment by Mendowski et al. (2020). ◆■ Samples from the experiment by Martin et al. (2021). ◆■ Samples from the experiment by Martin et al. (2023). ◆■ Samples from the experiment by Guyader et al. (2016). ◆■ Samples from the experiment by Edouard et al. (Unpublished). ◆■ Samples from the experiment by Edouard et al., (Unpublished). ◆■ Samples from the experiment by Bougouin et al. (2018) and Bougouin et al. (2019). ▲ Samples from the experiment by Edouard et al., (Unpublished). ▲ Samples from the experiment by Reynolds et al. (2016). ▲ Samples from the experiment by Manzocchi et al. (2023). For interpretation of the references to color in this figure legend, the reader is referred to the web version of this article.

The projections of the individuals in the first two principal components, as well as the projections of the supplementary variables, are shown in Figure 3. The first two principal components explain 50% of the variability. Both the individuals in the validation subset and those in the test subset are well represented by the individuals in the calibration subset.

The statistics for fecal NIR spectroscopy calibration, validation, and test data subsets for OMD prediction, obtained using the four approaches, are shown in Tables 4 and 5, respectively. In the models obtained from the calibration subset, the coefficient of determination for cross-validation (R²CV) varied between 0.58 for PLS regression and 0.66 for KNN-LWPLSR and KNN-LWPLSR-AGG, with the Locally weighted PLS regression (LWPLSR) showing an R²CV value of 0.65. The RMSECV ranged from 0.020 to 0.023 g/g.

**Table 4:** Calibration parameters and statistics for prediction of OM digestibility (g/g) according to the different Eq. developed: classic partial least square (PLS) regression, the Locally weighted PLS regression (LWPLSR), the k-nearest neighbors LWPLSR (KNN-LWPLSR) and the KNN-LWPLSR aggregated (KNN-LWPLSR-AGG).

	h	k^1^	Nlv	RMSECV	R^2^CV
**PLS**	–	299	12	0.023	0.58
**LWPLSR**	2	299	6	0.021	0.65
**KNN- LWPLSR**	3	200	6	0.020	0.66
**KNN-LWPLSR-AGG**	3	200	1 to 15	0.020	0.66

1 k: number of neighbors optimizing the prediction model; h: defines the weight function form; Nlv: number of latent variables; RMSECV: Root mean square error of cross-validation; R^2^CV: coefficient of determination of cross-validation.

**Table 5:** Validation and Test subset statistics for prediction of OM digestibility (g/g) according to the different Eq. developed: classic partial least square (PLS) regression, the Locally weighted PLS regression (LWPLSR), the k-nearest neighbors LWPLSR (KNN-LWPLSR) and the KNN-LWPLSR aggregated (KNN-LWPLSR-AGG).

	RMSEP	RMSEP(%)	Bias	RMSEP(C)	R^2^P	IP
**Validation subset**						
** PLS**	0.017	2.46	−0.002	0.017	0.80	±0.033
** LWPLSR**	0.016	2.35	−0.000	0.016	0.82	±0.031
** KNN- LWPLSR**	0.017	2.38	−0.001	0.017	0.81	±0.033
** KNN-LWPLSR-AGG**	0.016	2.30	−0.015	0.016	0.82	±0.031
**Test subset**						
** PLS**	0.019	2.67	−0.002b	0.019b	0.37	±0.037
** LWPLSR**	0.016	2.29	0.001a	0.016a	0.53	±0.031
** KNN- LWPLSR**	0.017	2.38	0.001a	0.017ab	0.50	±0.033
** KNN-LWPLSR-AGG**	0.017	2.39	−0.001a	0.017ab	0.49	±0.033

RMSEP: Root mean square error of prediction; RMSEP(%): Root mean square error of prediction expressed as percentage of the mean; RMSEP(C): Root mean square error of prediction corrected by the bias; R^2^P: coefficient of determination of prediction. IP: interval of model prediction. In each column, different letters indicate significant differences (*P* < 0.05).

Statistics from the validation subset (Table 5) were better (RMSEP were lower than RMSECV and R^2^P were higher than R^2^CV) than those of the calibration subset (Table 4). The coefficient of determination for prediction (R²P) ranged from 0.80 to 0.82, and the relative RMSEP values varied from 2.30 g/g for KNN-LWPLSR-AGG to 2.46 g/g for PLS regression. The bias values were all close to zero, and no significant differences (*P* > 0.05) were observed between calibration methods for either bias or RMSEP(C).

When the calibration models were applied to the test subset, the resulting statistics were lower than those obtained with the validation subset. Specifically, R²P values ranged from 0.37 to 0.53, and absolute and relative RMSEP values ranged from 0.016 g/g or 2.29 % to 0.019 g/g or 2.67 %, respectively. Despite these differences, the bias for all models remained close to zero even though significant differences (*P* < 0.05) were found between the Local methods and the PLS model. Notably, the RMSEP(C) values for the LWPLSR and KNN-LWPLSR-AGG models were significantly lower than those for the PLS regression model. No significant differences were found between the Local PLS regression models.

## Discussion

The fecal samples used in the present study were collected from cows exhibiting a moderate range of OMD values, spanning from 0.60 to 0.78 g/g, comparable to similar studies in the literature (Boval et al. 2004; Jancewicz et al. 2016; Peters et al. 2023). These samples were obtained from animals with varying nutritional needs, including dry cows fed exclusively on grass hay and high milk-yielding dairy cows receiving total mixed rations formulated to provide high levels of metabolizable energy and protein (Table 1, see references). Despite this broad variability, the coefficient of variation (CV = 4.9%) observed in our study was lower than the 8.6% CV reported by Andueza et al. (2017) for OMD determination in temperate forages fed to castrated male sheep. While most factors influencing the nutritive value of animal diets are represented in our dataset, it is important to note that the number of beef cows included is relatively low compared to the number of dairy cows. Among the dairy cows, the only breed used was Holstein Jancewicz et al. 2017 (see Table 1).

The projections of the supplementary variables on the PCA score plot of the first two principal components show that PC1 is explained by the type of forage consumed by the animals, while PC2 is not explained by the tested supplementary variables. The treatments evaluated within each study could partially explain this PC. The spectra obtained in the present study come from samples ground to two particle sizes, 0.8 and 1 mm. In addition, two different types of mills were used to grind the samples: a hammer mill and a cyclone mill (Table 1). These factors could, firstly, negatively influence the prediction results of the prediction models. However, data pre-processing can help correct these negative effects. On the contrary, including values obtained under different conditions in the same database can contribute to increasing the robustness of the database. Both variables (particle size and type of mill) have little influence in explaining the variability observed in the first two PCs (Figure 3). The majority of the samples collected for the present study were obtained from dairy cows. A total of 64 samples were collected from beef cows (Table 1; de la Torre et al. 2019). The positioning of these samples on the biplot (in close proximity to the center) suggests that their incorporation into the database exerts minimal influence on the elucidation of the variability observed in the first two PCs (Figure 3).

In the current study, the standard error of the reference method was estimated to be 0.0135 g/g, based on data from de la Torre et al. (2019). This value closely aligns with the 0.010 g/g reported by Andueza et al. (2011) for estimating the standard error of OMD in forages using castrated male sheep aged 2 to 4 years with a mean live weight of 60 kg. In a recent study by Bellagi et al. (2025), dry matter digestibility was measured twice per animal in fattening young bulls fed either low- or high-nitrogen diets. The resulting standard error—based on a measurement duration comparable to that of the present study—was closely aligned with ours, ranging from 0.0114 to 0.0153 g/g. The value of 0.0135 g/g here obtained represents the minimum possible error that could be expected from any of the prediction models, as all models are based on values derived from reference methods. As such, it serves as a benchmark for evaluating the accuracy of our prediction models in the present work.

The cross-validation R^2^ value of 0.58 for the models obtained in this study is relatively low compared to the PLS regression model reported by Andueza et al. (2017), which achieved an R^2^CV of 0.86 for OMD of forages using castrated male sheep (mean of 6 animals), that of Boval et al. (2004), who reported a R^2^CV​ of 0.69 for OMD of tropical grass ingested by Creole cattle, and those of Jancewicz et al. (2016) and Peters et al. (2023). These authors report R^2^V values of 0.80 and 0.65 in their respective studies on beef cows. However, when the RMSCV values are expressed as a proportion of the mean OMD value, the PLS regression models from all studies yield similar results: 3.29% for Andueza et al. (2017), 3.13% for Boval et al. (2004), 4.30% for Jancewicz et al. (2016), 3.52% for Peters et al. (2023), and 3.24% for the current study. The broader range of OMD values in the datasets of these authors mentioned may account for the higher R^2^ values reported in those studies compared to the value observed in the present study.

The cross-validation results were higher than the RMSEP obtained through the validation and test subsets. According to Esbensen and Geladi (2010), cross-validation is a suboptimal simulation of validation because it does not account for sampling variance, as it relies on a single dataset (the calibration subset). However, when comparing the coefficients of determination between the validation subset and the test subset, the results were also distinct, with R^2^ values of around 0.80 for the validation subset and 0.50 for the test subset. Westad and Marini (2015) explain that the Kennard-Stone method aims to capture the maximum diversity of samples in the calibration subset, which may lead to overoptimistic results, as observed in our study. Nevertheless, when comparing the RMSEP values expressed as a proportion of the mean, the differences between the validation and test subsets were smaller than those seen for the coefficients of determination, particularly for the three Local models. The three Local models outperformed the classic PLS regression, in line with the findings of Andueza et al. (2017). This supports the notion that feces are a heterogeneous biological material subject to nonlinear variability.

In the current study, no significant differences were observed between the various Local regression methods tested, contrary to our hypothesis, which was that KNN-LWPLSR-AGG would perform better than LWPLSR and KNN-LWPLSR based on the findings of Lesnoff et al. (2020). These authors, working with different databases of chemical composition and nutritive value of forages, reported improved results when averaging methods were employed. However, these averaging methods are characterized by more time-consuming calibration processes compared to the LWPLSR and KNN-LWPLSR methods tested in the present study.

Based on the prediction interval obtained, our NIR prediction Eq. can distinguish between two individual observations if their OMD values differ by at least 0.06 g/g (Interval of prediction = ±0.031; Table 5) corresponding to the minimum detectable difference of the prediction. From a practical perspective, this indicates that NIR can effectively differentiate between two animals fed substantially different diets. However, considering the variability observed between animals consuming the same diet, these results suggest that NIR predictions are less reliable for detecting differences between individual animals. However, it is important to contextualize these findings, as the prediction error interval derived from the reference method indicates that a minimum detectable difference of 0.05 g/g (calculated as 2 × 1.96 × 0.0135 g/g) can be detected. Practically, this value is comparable to those obtained from the NIR predictions. Additionally, it is important to note that in vivo digestibility trials are considerably more expensive but also invasive, time-consuming, and heavy-workload than NIR-based predictions. As a result, using NIR predictions enables a substantial increase in the number of animals that can be phenotyped, even with slightly reduced accuracy. To further explore this, we conducted a statistical power test using our RMSEP to calculate the minimum number of animals required to detect a significant effect on OMD. For example, our analysis showed that the significant difference in OMD observed by Pirondini et al. (2015) (*P* = 0.02), when comparing dairy cow diets with or without fish oil supplementation (effect size: 0.024 g/g, or 3.5% CV), could have been detected using fecal samples from approximately 12 (with LWPLSR model) to 16 (with classical PLS regression model) animals per treatment, while maintaining the same significance level. This number is slightly higher than the 8 animals per group used in their original Exp.

One practical limitation of this study is that the OMD predictions were based on fecal samples derived from 24-h total collections conducted over several days. Due to the lack of spot samples in most of the trials used, it was not possible to assess whether spot-collected samples—which are more practical to obtain under field conditions—would yield comparable predictive performance. It is well established that substantial variability exists among spot samples collected from the same animal at different times or on different days (Jancewicz et al. 2017; Giagnoni et al. 2021). This temporal variability might suggest that calibration models developed using spot samples would perform less reliably than those based on total collection samples, as used in this study. Future research should investigate the feasibility of using spot samples as a practical alternative for implementing this approach in real-world field conditions.

The use of local models in practice could simplify spectral data management, as only the spectral database needs to be maintained. In contrast, traditional NIR approaches require the maintenance of one or more spectral databases in addition to the periodic updating of calibration models. One potential limitation of local PLSR approaches is their computational cost (Lesnoff et al. 2020). However, the algorithms evaluated in this study are not excessively time-consuming. Among them, the KNN-LWPLSR-AGG algorithm requires more computation time than LWPLSR and KNN-LWPLSR, without consistently improving prediction performance. Considering both prediction accuracy and computational cost, LWPLSR appears to offer a suitable compromise between model performance and computing time.

## Conclusions

This study demonstrates the potential of fecal NIRS models to predict OM digestibility in cattle exhibiting a moderate range of OMD values. The implementation of Local modeling approaches significantly reduced prediction errors (RMSEP), highlighting their effectiveness in improving model performance. Among the Local methods evaluated, LWPLSR is recommended due to its balance between predictive accuracy and computational simplicity. Our best model (RMSEP = 0.016) has the potential to predict OM digestibility in cattle provided that a slightly larger sample size is used compared to reference in vivo methods. Further research is warranted to evaluate the predictive performance of these models using spot-collected fecal samples to ensure their feasibility for widespread use in practical, on-farm conditions.

## Data Availability

The data underlying this article will be shared on reasonable request to the corresponding author.

## Abbreviations

A: animal
CV: coefficient of variation
D: diet
d: effect size
h: weight function parameter
H: Mahalanobis distance
k: number of nearest neighbors
KNN-LWPLSR: k-nearest neighbors Locally weighted partial least square regression
KNN-LWPLSR-AGG: k-nearest neighbors Locally weighted partial least square regression with aggregation
LWPLSR: Locally weighted partial least square regression
NIR: near infrared
NIRS: near-infrared reflectance spectroscopy
nlv max: maximum number of latent variables
Nlv min: minimum number of latent variables
OM: organic matter
OMD: organic matter digestibility
P: period
PLS: partial least squares
R^2^: coefficient of determination
R^2^CV: coefficient of determination in cross-validation
R^2^P: coefficient of determination of prediction
RMSE: root mean square error
RMSECV: root mean square error of cross-validation
RMSEP(C): root mean square error of prediction corrected for bias
RMSEP: root mean square error of prediction
Z_[1-β]_: statistical power
Z_α_: standard normal deviates corresponding to the Type I error
